# Surface molecular structure defects and laser-induced damage threshold of fused silica during a manufacturing process

**DOI:** 10.1038/s41598-017-18249-2

**Published:** 2017-12-19

**Authors:** Yuan Li, Hongwei Yan, Ke Yang, Caizhen Yao, Zhiqiang Wang, Xinshu Zou, Chunyan Yan, Xiaodong Yuan, Xin Ju, Liming Yang

**Affiliations:** 1 Department of Physics, University of Science and Technology Beijing, Beijing, 100083 China; 2 Laser Fusion Research Center, China Academy of Engineering Physics, Mianyang, 621900 China; 3 Fine Optical Engineering Research Center, Chengdu, 610041 China

## Abstract

Laser induced damage of fused silica is a serious problem for high power laser systems, and damage precursors are mainly induced by manufacturing processes. In this work, fused silica samples were prepared from a manufacturing process including grinding, polishing and etching procedures. The chemical disorder of the prepared samples was inspected by using fluorescence microscopy and ultra-violet fluorescence spectrometer. The physical disorder was characterized by using Infrared and Raman spectrometer. Laser induced damage thresholds (LIDTs) were measured in R-on-1 mode by 355 nm 6.4 ns laser pulse. Results showed that with the manufacturing processes transforming from grinding to etching, the magnitude of fluorescence point defects reduced while their types did not change, the Si-O-Si bonds of prepared samples were strained and the strained bonds were mitigated. The LIDTs increased with the reducing of fluorescence defects and strained Si-O-Si bonds. However, these structural defects can not be eliminated by the current manufacturing process. Improvements may be needed to eliminate the structural defects for a higher LIDT of fused silica.

## Introduction

Fused silica optics are widely used in high power laser systems such as National Ignition Facility (NIF) and Laser Megajoule (LMJ)^1,2^. When subjected to 355 nm laser pulse irradiation at high fluence, fused silica optics may suffer from laser induced damage^3–5^. Inpurities and fracture defects were found to be the main damage precursors which were responsible for the initial of laser damage^6,7^. These damage precursors were usually induced in manufacturing process, and can be mitigated by the improvement of the manufacturing technologies. Recently, laser damage initiation density has been reduced significantly by using improved fabrication processes and the advent of whole optics mitigation strategies^8–11^. Based on the elastic-plastic deformation theory, Shi feng *et al*.^12^ analyzed status of abrasives and workpiece in magnetorheological finishing (MRF) process and the feasibility of elastic polishing. Results showed that laser induced damage threshold (LIDT) was improved from 9.77 to 19.2 J/cm^2^ after MRF elastic polishing. J. Bude *et al*.^13^ reduced damage density in fused silica at high fluence (>40 J/cm^2^) by more than 100 times by minimizing the presence of precipitates during chemical etching process. P. E. Miller *et al*.^6^ combined MRF polishing and chemical leaching to explore the defects influence on the damage of fused silica and found that a thin defect layer on fracture surfaces is the dominant source of laser damage initiation.

Though the LIDTs of fused silica optics have been successfully improved, it is still far below the intrinsic threshold (~150 J/cm^2^) of fused silica^14,15^. While, the damage precursors are still quite vague at the fluence above ~40 J/cm^2^. It is difficult to locate impurities in nanoscale on a sample surface with a size of several centimeters. While, a tiny variation of molecular structures can be detected by a fluorescence, infrared or Raman spectroscopies. Fused silica may contain two types of structural defetcs: chemical disorder (point defetcs) and physical disorder (Si-O-Si bond angle and (Si-O)_n_ ring structure)^16,17^. In this work, efforts were made to inspect surface molecular structures of fused silica samples during a manufacturing process. The influence of molecular structural defects (chemical and physical disorders) on LIDTs of fused silica optics were explored.

## Results and Disscusion

### Chemical disorder

**Figure 1:**
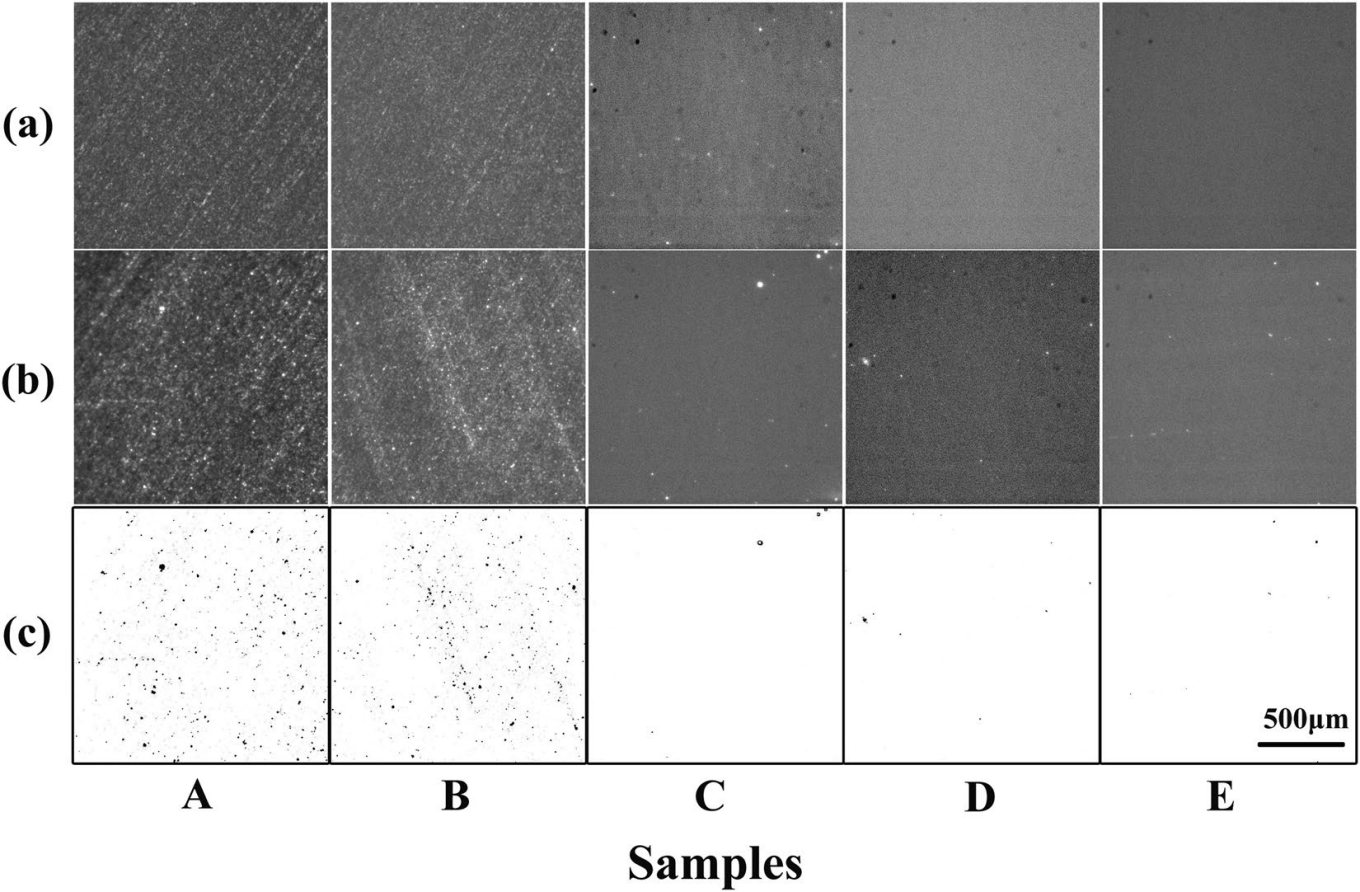
Images of samples: (**a**) bright field images, (**b**) fluorescence images and (**c**) inversed images. All images share the same scale bar. (Please refer to the methods section for processing parameters of samples A, B, C, D, and E).

**Figure 2:**
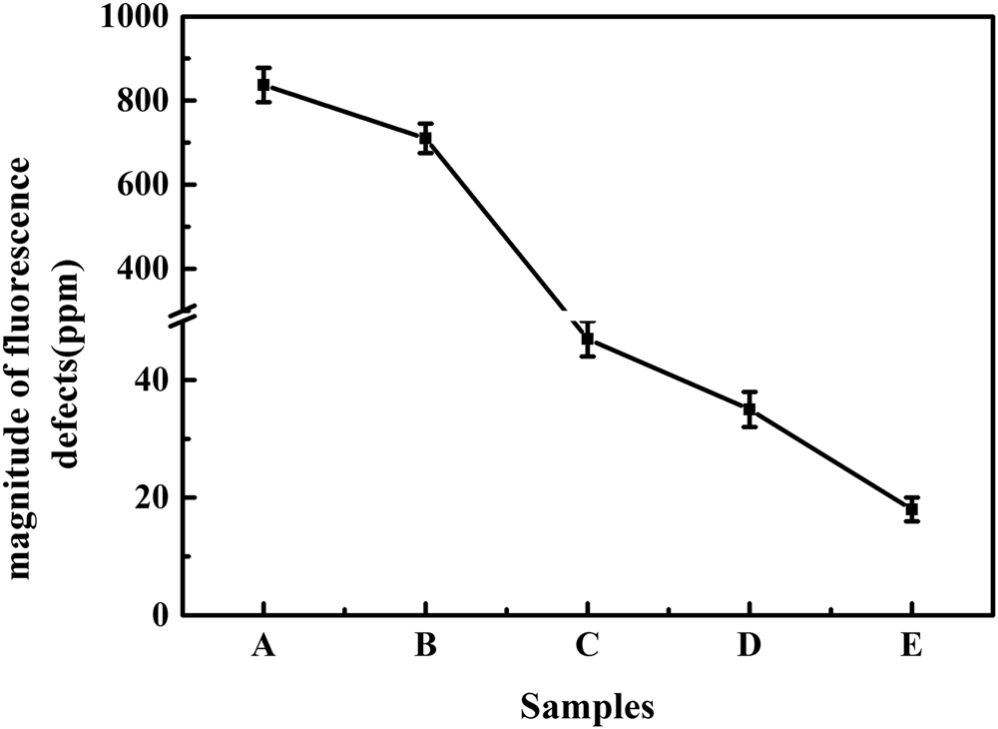
fluorescence defects magnitude of treated samples. The unit of ppm refers to part per million.

Figure 1 shows bright field images (a), fluoresence images (b), and inversed images (c) of prepared samples. Samples A and B were ground by different size of abrasive. Samples C and D were polished by ceria and MRF polishing, respectively. Sample E was prepared by MRF polishing and HF etching. In the bright field images, the bright points indicated the scattering lights due to the surface fractures induced by the processing. While the bright points in the fluoresence images were corresponding to the fluorescence by the surface fractures. It is obvious that surface fractures of ground samples are more than those of polished samples, and the etched sample possesses less fractures than the polished samples. To quantify the magnitude of fluoresence points in these images, the figures of Fig. 1(b) were transformed to gray value images (range of the value, −255~255 (a.u.)) and then filtered by the background (value of 100 (a.u.), in our situation). The contrast was inversed to give a better visual effect, as shown in Fig. 1(c), and the black points represented the positions where the fluoresence occured. As shown in Fig. 2, the surface fluorescence defects magnitude of ground samples A and B is about one order more than those of other samples.With the precedures transforming from grinding to etching, the surface fluorescence defects magnitude reduced.

**Figure 3:**
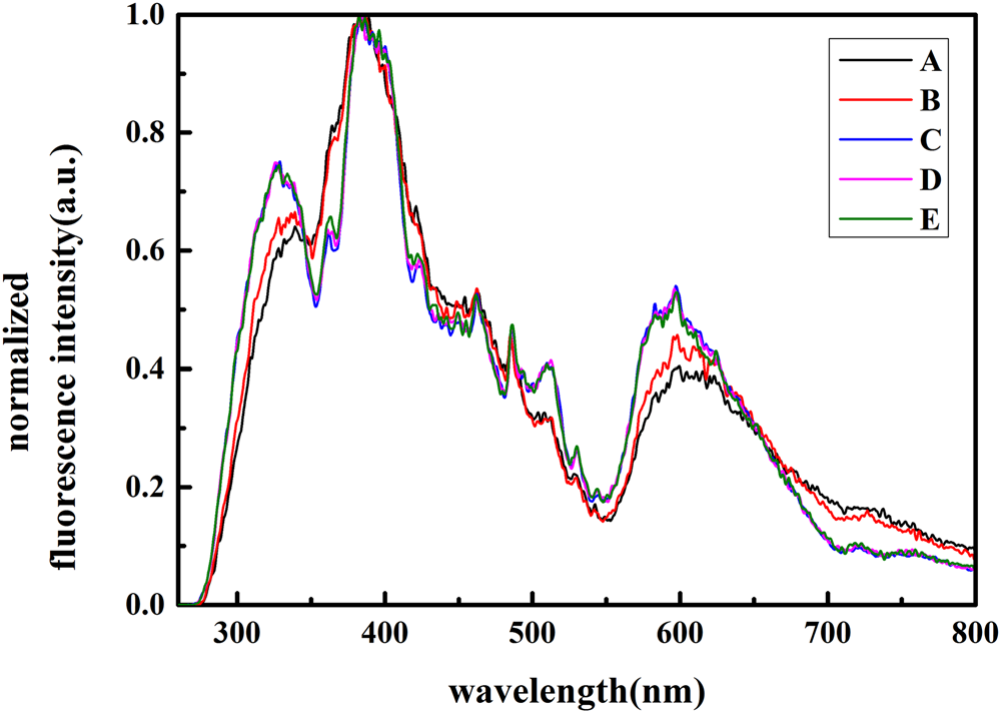
The normalized fluorescence emission spectra of samples.

Figure 3 shows the comparison of normalized fluorescence emission spectra of treated samples. There are three main peaks in the spectra at around 310 nm, 390 nm and 590 nm bands, corresponding to oxygen-deficiency centers (II) (ODC(II), around 290 nm for α band and 390 nm for β band), and *E*_*δ*_’ center defects (590 nm band), respectively^18–20^. The characterized peak at around 310 nm band is an incomplete peak of ODCs (around 290 nm band) due to the application of filter (290 nm).

In the gringding procedures, the decrease of abrasive particles size hardly changed the species and the relative ratios of the fluorescence defects. The polished and etched samples possessed the same defects species with the ground samples. However, the relative ratios of 310 nm and 590 nm defects were higher than those of ground samples. In other words, though the improvement of the manufacturing procedures, the types of fluorescence defets were remained the same while the relative ratio of 390 nm defect was reduced. The fluorescence defects with lifetime shorter than the laser pulse correlate to damage propensity by the single-photon absorption of sub-band gap light^21^. According to L. Skuja *et al*.^18,20,22^, the lifetimes of these fluorescence defects discussed above are 4 ns (α-ODC(II)), 110 μs (β-ODC(II)) and 300 ns (*E*_*δ*_’), respectively. In our case (laser pulse is 6.4 ns), α-ODC(II) might be the main laser damage precursor. Unfortunately, it was not removed by the current manufacturing processes. However, a detailed understand about this 310 nm defect is still open to the further work.

### Physical disorder

**Figure 4:**
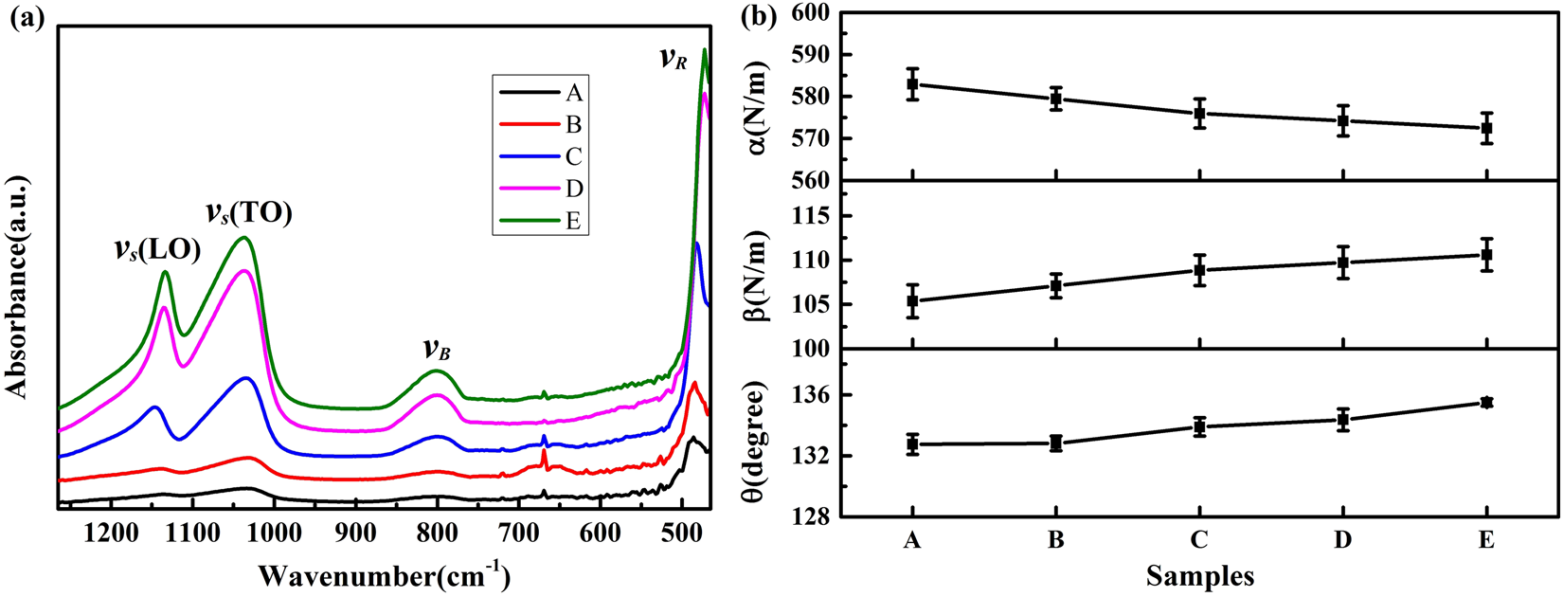
Infrared absorption spectra of samples (**a**) and the calculated structure parameters of the Si-O-Si bonds (**b**).

The infrared spectra are carried out to find the strained Si-O-Si bonds of the prepared samples surfaces as shown in Fig. 4. Under the irradiation of ns laser, the strained Si-O-Si bond is an absorptive precursor, which induces damages and leads to the reduction of laser damage threshold of fused silica. These spectra typically include four peaks corresponding to four vibrational modes of Si-O-Si bond: *ν*_*R*_ (rocking mode, ~470 cm^−1^), *ν*_*B*_ (bending vibration, ~800 cm^−1^), *ν*_*S*_ **(** *TO* **)** (transverse optical mode of asymmetric stretching vibration, ~1020 cm^−1^), and *ν*_*S*_ **(** *LO* **)** (longitudinal optical mode of asymmetric stretching vibration, ~1130 cm^−1^)^23^. The wavenumbers of these peaks can be theoretically derived by using a single nearest neighbor central force assumption^24^ with a short-range Born potential^25^, and can be calculated by the following equations^26,27^:

1 $${v}_{R}=\frac{1}{2\pi c}\sqrt{\frac{2\beta }{{m}_{O}}}$$

2 $${v}_{B}=\frac{1}{2\pi c}\sqrt{\frac{4{\boldsymbol{(}}\alpha +2\beta {\boldsymbol{)}}}{3{m}_{Si}}}$$

3 $${v}_{S}=\frac{1}{2\pi c}\sqrt{\frac{2}{{m}_{O}}(\alpha \,si{n}^{2}\frac{\theta }{2}+\beta \,co{s}^{2}\frac{\theta }{2})}$$

In which, *α* is the central force constant, *β* is the non-central force constant, and *θ* is the Si-O-Si bond angle. m_Si_ and *m*_*O*_ are the atomic mass of silicon and oxygen, respectively. *c* is the velocity of light. The force constants (*α* and *β*) are related to elastic constants: *α* determines Young’s modulus, and *β* determines the shear modulus^26^. The angle of Si-O-Si bond (*θ*) is related to the density of surface materials^28^. These structure parameters can be derived from the wavenumbers of the infrared spectra by using equations mentioned above.

The calculated values of structure parameters are shown in Fig. 4(b). The central force constant values of the polished samples are higher than those of grinded samples and lower than those of etched samples (*α*_grinded_ < *α*_polished_ < *α*_etched_). The tend of the non-central force constant is opposite (*β*_grinded_ > *β*_polished_ > *β*_etched_). Young’s modulus increases with decreasing *α*, while shear modulus decreases with decreasing *β*. Thus, Young’s modulus and shear modulus of grinded samples are higher than those of polished samples, and the etching process make the elasticity moludus smaller.

The estimated Si-O-Si bond angles (*θ*) are also shown in Fig. 4(b). The angles of all prepared samples are lower than the average value estimated by X-ray diffraction (~145°)^29,30^. This discrepancy indicates that Si-O-Si bonds in the surface layer are strained and the densities of the surface materials after grinding, polishing and etching are slightly higher than that of the body material of fused silica. The densification of surface materials may due to the normal pressure employed during the grinding and polishing processes.

**Figure 5:**
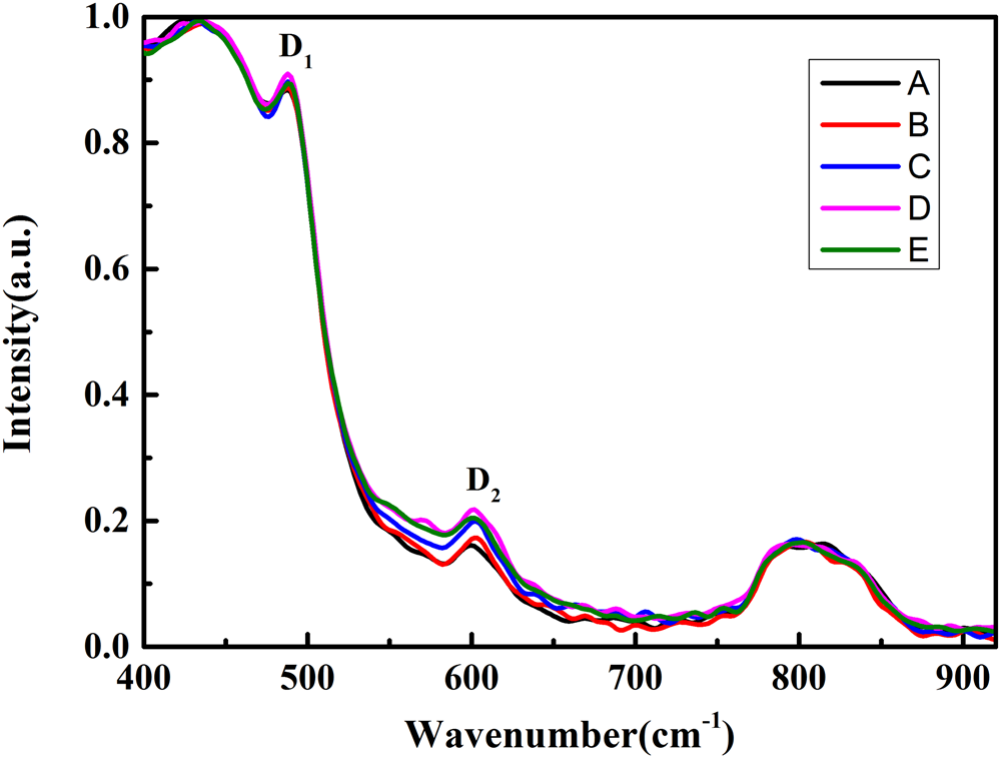
The Raman spectra of samples. D_1_ and D_2_ indicate the four- and three- member rings of SiO_4_ tetrahedra, respectively.

Raman spectra of treated samples are shown in Fig. 5. The two sharp contributions at 490 cm^−1^ (D_1_) and 606 cm^−1^ (D_2_) correspond to the symmetric stretching vibrations (“breathing mode”) in four- and three- member rings of SiO_4_ tetrahedra^31^. Si-O-Si bonds in these smalll ring structures are strained and may result in defect pairs of *E’* and NBOHC under the laser irradiation^16^. Results of both Infrared and Raman spectra indicate that the current manufacturing processes can reduce the strained Si-O-Si bonds but still can not eliminate them.

### LIDTs

**Figure 6:**
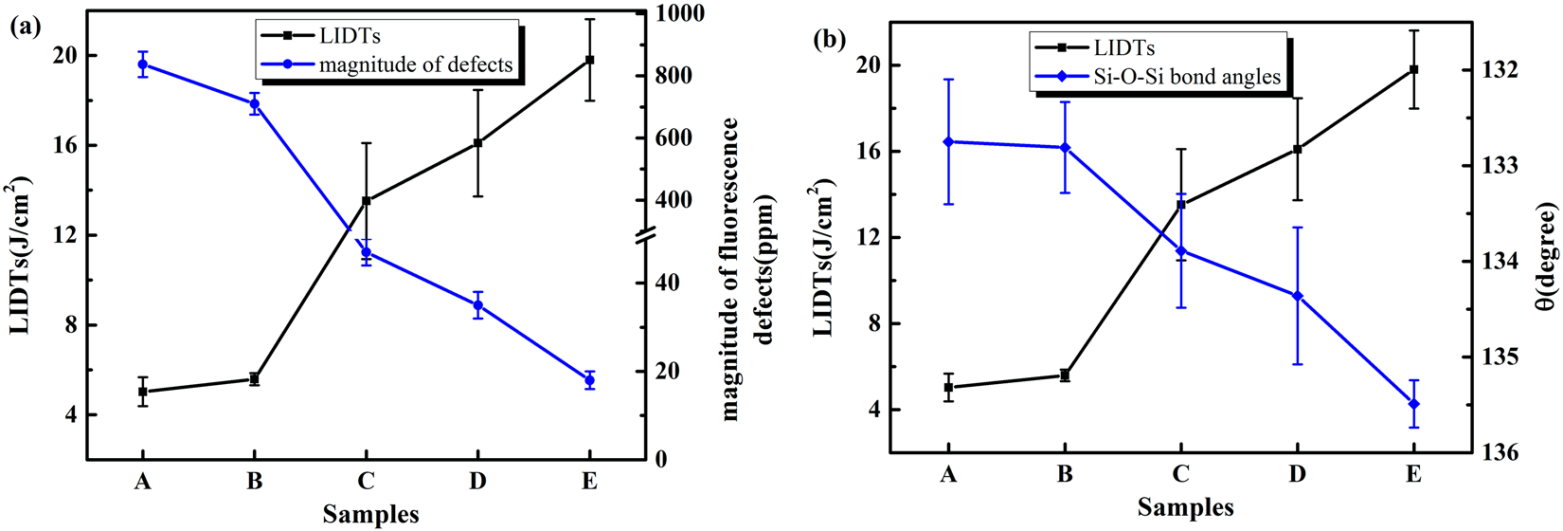
Comparison of R-on-1 LIDTs of the samples, and the influence of fluorescence defects and Si-O-Si bonds on LIDTs of fused silica optics.

The R-on-1 LIDTs of samples are shown in Fig. 6. The LIDTs of grinded samples are 5.0 J/cm^2^ and 5.6 J/cm^2^, which are less than those of polished samples (13.5 J/cm^2^ for ceria polishing and 16.1 J/cm^2^ for MRF finishing, respectively). The etched Sample E has the highest LIDT of 19.8 J/cm^2^. Combining with the discussions above of fluorescence defects and strained Si-O-Si bonds, the sample with more fluorescence defects and strained Si-O-Si bonds has a lower LIDT, as seen in Fig. 6.

The effect of abrasive particle size may due to the difference of average load on per particle^8^. The average load grows with the increase of abrasive particle size, and the fractured cracks occur under high average load during grinding process. Consequently, for samples A and B, the usage of smaller abrasive particles brings less fluorescence defects and strained Si-O-Si bonds. It has been reported that the MRF process can mitigate surface and subsurface defects of conventional polished samples while leave a thin surface Beilby layer^32,33^, which can be mitigated after etching process. However, the fluorescence defects and strained Si-O-Si bonds still exist and limit the LIDT of fused silica optics.

## Conclusions

This paper presents fluorescence defects, surface molecular structures and laser damage performances of fused silica samples prepared by grinding, polishing and etching processes. Results showed that the fluorescence defects and strained Si-O-Si bonds reduced with the improvement of the manufacturing process from gringing to etching and LIDTs of fused silica increased with the reducing of the fluorescence defects and strained Si-O-Si bonds. However, these structural defects can not be eliminated by the manufacturing process disscused here, and limited the LIDT of fused silica under 6.4 ns 355 nm laser irradiation. Both the grinding and polishing processes will induce fluorescence defects and strained Si-O-Si bonds to the surfaces of fused silica samples. Though the HF acid etching process can mitigate thoses precursors and improve the LIDTs of fused silica samples, but improvements are still needed to eliminate these structural defects for a higher laser damage resistance of fused silica.

## Methods

### Sample preparation

Fused silica samples (50 mm × 50 mm × 10 mm; Corning 7980, Corning, NY) were firstly polished down to 1 nm (RMS) on both sides. One face of each sample was then treated by one of the manufacturing procedures with 1.5 μm removed:

- Sample A was ground on a Strasbaugh Model 6Y2 grinder using 15 μm Al_2_O_3_ abrasive in water on a Pyrex glass lap (load = 25 N, lap rotation rate = 16 rpm).
- Sample B was ground on a Strasbaugh Model 6Y2 grinder using 9 μm Al_2_O_3_ abrasive in water on a Pyrex glass lap (load = 25 N, lap rotation rate = 16 rpm).
- Sample C was conventionally polished by continuous pitch polishing techniques using 10 μm ceria (CeO_x_) abrasive.
- Sample D was subjected to MRF polishing using standard ceria (CeO_x_) slurry contained in an iron based MR fluid media.
- Sample E was firstly polished by the same MRF polishing as the Sample D, and then etched in 5%wt HF etchant with ultrasonic agitation of 40–270 kHz for 5 min to remove the surface Beilby layer.

Before LIDT tests, all samples were clearned in ultrapure water with ultrasonic agitation of 40–270 kHz for 30 min, and allowed to air dry in a clean room.

### R-on-1 LIDTs

**Figure 7:**
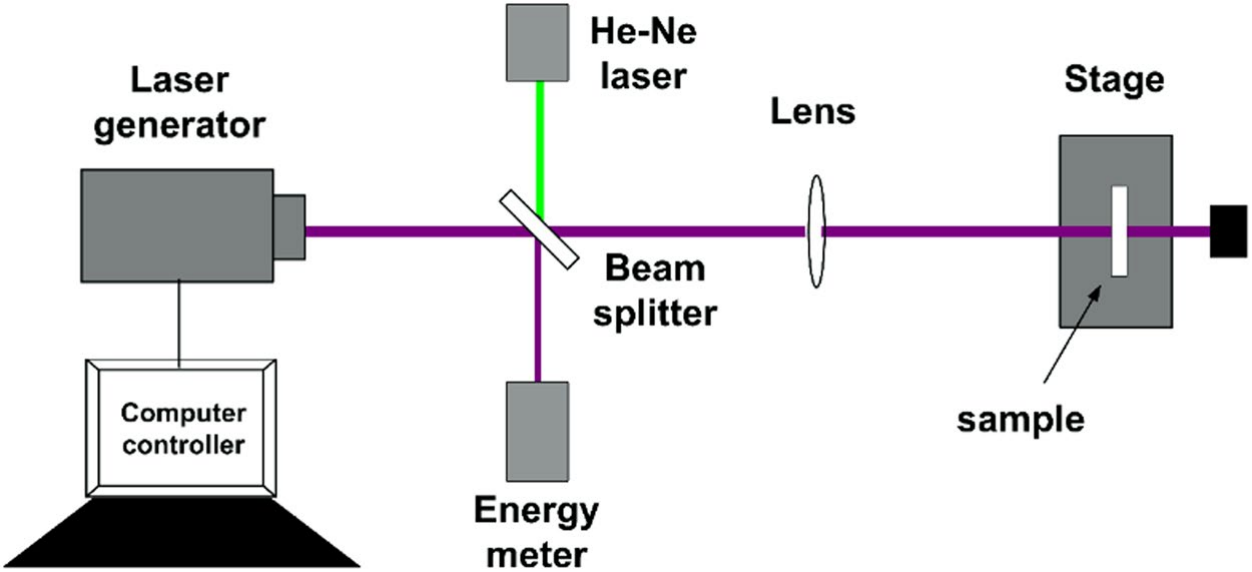
Schematic of the experimental setup for laser damage tests.

R-on-1 LIDTs tests were carried out at ambient conditions by using a 6.4 ns 355 nm beam from a Q-switched Nd-YAG laser. The beam profile was Gaussian with a 1/e^2^ area of 0.6 mm^2^ at the sample plane. The test laser fluence was started at 1 J/cm^2^ and then increased by 0.1 J/cm^2^ each shot until the damage occurred (determined by *in situ* optical microscopy). Schematic of the experimental setup for laser damage tests is shown in Fig. 7. The surfaces prepared by different procedures were rear surfaces of samples.

### UV confocal fluorescence image

An integrated confocal fluorescence microscope system consists of a fluorescence microscope, laser light sources, and a scan head. The laser was directed on the sample and the emission was collected, a computer with software was used to control the scan head and display the acquisition. The excitation beam was 355 nm laser and the images were 20 × objective. Both fluorescence and bright field images can be obtained.

### PL spectra

PL spectra were carried out on a fluorescence spectrometer equipped with a photomultiplier detector. Excitation was realized at 248 nm by a Xe lamp at ambient temperature. The focal spot size on the sample was about 50 mm^2^, and the slit width for exitation and emission was 7 nm and 4 nm, respectively. A 290 nm high-pass filter was laid in front of the detector.

### Infrared spectra

Fourier transform infrared absorption spectra were obtained by a Nicolet 5700 spectrometer. A reflection mode was employed to measure the spectra in a frequency region of 400–1300 cm^−1^, associating with the Si-O-Si stretching, bending, and rocking vibrations. The depth of penetration into the sample is in the order of a few micrometers. All spectra were taken at ambient temperature with more than 200 scans at a resolution of 0.96 cm^−1^.

### Raman spectra

Raman spectra were obtained with more than 300 scans at ambient conditions by using the Nicolet spectrometer. The spectral resolution was ~0.96 cm^−1^, and the spot diameter was 50 μm.

### Data availability statement

All data generated or analysed during this study are included in this published articl.
